# Cell types differ in global coordination of splicing and proportion of highly expressed genes

**DOI:** 10.1038/srep32249

**Published:** 2016-08-31

**Authors:** Ephraim F. Trakhtenberg, Nam Pho, Kristina M. Holton, Thomas W. Chittenden, Jeffrey L. Goldberg, Lingsheng Dong

**Affiliations:** 1 Department of Neurosurgery, F.M. Kirby Neurobiology Center, Boston Children’s Hospital, Harvard Medical School, Boston, MA, USA; 2 Research Computing Group, Harvard Medical School, Boston, MA, USA; 3 Byers Eye Institute, Stanford University, Palo Alto, CA, USA

## Abstract

Balance in the transcriptome is regulated by coordinated synthesis and degradation of RNA molecules. Here we investigated whether mammalian cell types intrinsically differ in global coordination of gene splicing and expression levels. We analyzed RNA-seq transcriptome profiles of 8 different purified mouse cell types. We found that different cell types vary in proportion of highly expressed genes and the number of alternatively spliced transcripts expressed per gene, and that the cell types that express more variants of alternatively spliced transcripts per gene are those that have higher proportion of highly expressed genes. Cell types segregated into two clusters based on high or low proportion of highly expressed genes. Biological functions involved in negative regulation of gene expression were enriched in the group of cell types with low proportion of highly expressed genes, and biological functions involved in regulation of transcription and RNA splicing were enriched in the group of cell types with high proportion of highly expressed genes. Our findings show that cell types differ in proportion of highly expressed genes and the number of alternatively spliced transcripts expressed per gene, which represent distinct properties of the transcriptome and may reflect intrinsic differences in global coordination of synthesis, splicing, and degradation of RNA molecules.

How does a cell maintain global properties of the transcriptome? This question has been addressed using thermodynamic models explaining the maintenance of RNA homeostasis and involving equilibrium between synthesis and degradation^1^,^2^,^3^,^4^,^5^,^6^,^7^,^8^,^9^. Evidence also exists that global levels of transcription could be affected by genes such as c-Myc or by chromosomal aneuploidies^10^,^11^,^12^, however, it is unknown whether various mammalian cell types differ intrinsically in how they maintain their global properties of the transcriptome. For example, do different cell types vary in a negative feedback threshold or a general molecular mechanism for regulating the levels of highly expressed genes? Is alternative splicing mechanism active at similar levels across cell types?

To investigate these questions, we compared proportion of expressed genes, alternatively spliced transcripts, and other global properties of the transcriptome at different expression thresholds in transcriptome profiles of 8 purified mouse cell types from different developmental lineages: retinal ganglion cells (RGC)^13^, cortical neurons, astrocytes, oligodendrocytes, microglia, endothelial cells^14^, megakaryocyte-erythroid progenitors (MEP), and erythroid-committed precursors (ECP) Gata1 knockout (KO, which cannot differentiate into the erythroid cells without Gata1)^15^,^16^.

## Results

**Figure 1:**
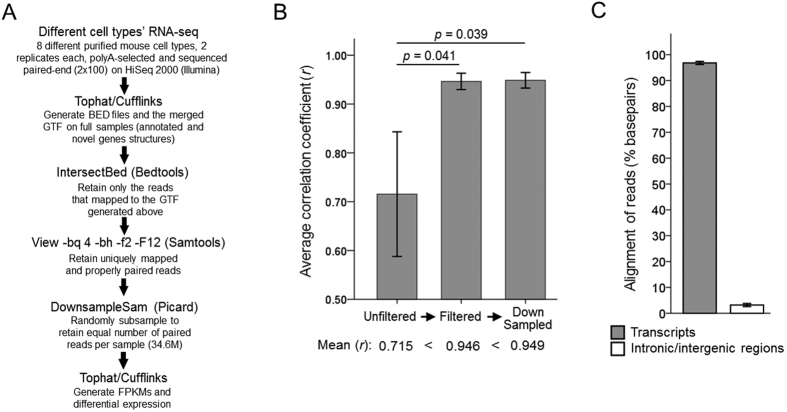
Preparation of datasets for analysis. (**A**) Data filtering and gene expression analysis pipeline. (**B**) Mean correlation coefficient between replicates within the samples increases after filtering and subsampling (Pearson *r*, 2-tailed; *n* = 8; *p*-values by ANOVA with posthoc LSD). (**C**) On average over 95% of the filtered reads aligned to transcripts across cell types, with less than 5% percent aligning to introns and intergenic regions (*n* = 8, mean ± SEM of basepairs aligned to transcripts or introns/intergenic regions shown as percent of total aligned basepairs; alignment percent determined by Picard module RnaSeqMetrics).

To analyze the cell types’ transcriptome profiles, we selected the datasets that had two replicates and were generated using libraries prepared from the polyA-selected RNA and paired reads sequenced 100 bp from each end on HiSeq 2000 Sequencer (Illumina) in all samples. The origins of the datasets used in this study are shown in Table 1. We analyzed the datasets using the Cufflinks pipeline^17^,^18^,^19^ (class codes for the novel predicted transcripts are summarized in Figure S1). As comparative RNA-seq analyses could be affected by noise, sequencing depth, gene length, and normalization^20^,^21^,^22^,^23^,^24^,^25^, we filtered the datasets to improve their quality (the pipeline is summarized in Fig. 1A; see Methods for details). Filtering improved quality of the data, as shown by average correlation between replicates within the samples increasing from *r* average of 0.715 in unfiltered to 0.946 in filtered, and further to 0.949 after random subsampling (Fig. 1B). The filtered replicates’ gene expression profiles were highly correlated within but not between the samples (correlation matrix in Table 2). On average over 95% of the filtered reads aligned to transcripts across cell types, with less than 5% percent aligning to introns and intergenic regions (Fig. 1C).

**Figure 2:**
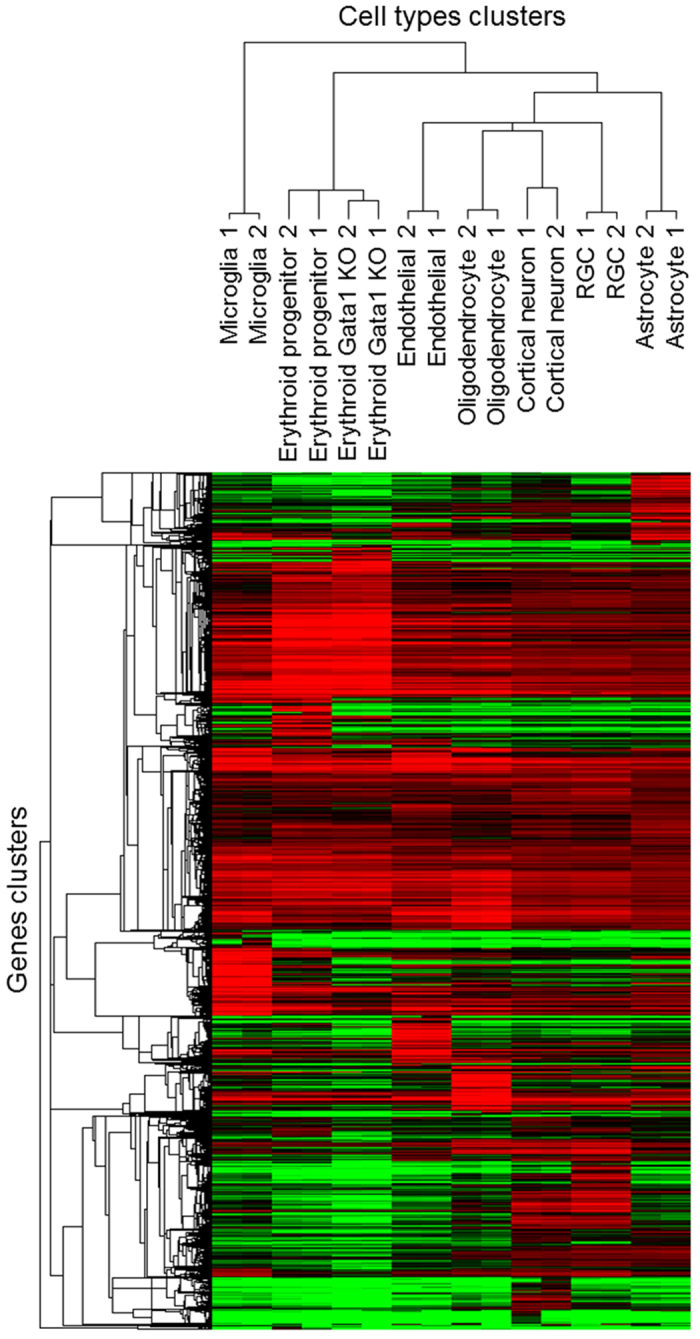
Clustering and heat map of cell types’ gene expression profiles. Dendrogram and unsupervised hierarchical clustering heat map of cell types (2 replicates each), using uncentered Pearson correlation and centroid linkage. The vertical distances on each branch of the dendrogram represent the degree of similarity between cell types’ gene expression profiles. 18,439 genes expressed above 1 FPKM in at least one sample were analyzed with Gene Cluster 3.0 and visualized with Java Treeview 1.1.6r4 (expression level is color coded: red for over-expressed, black for unchanged expression, and green for under-expressed genes).

**Figure 3:**
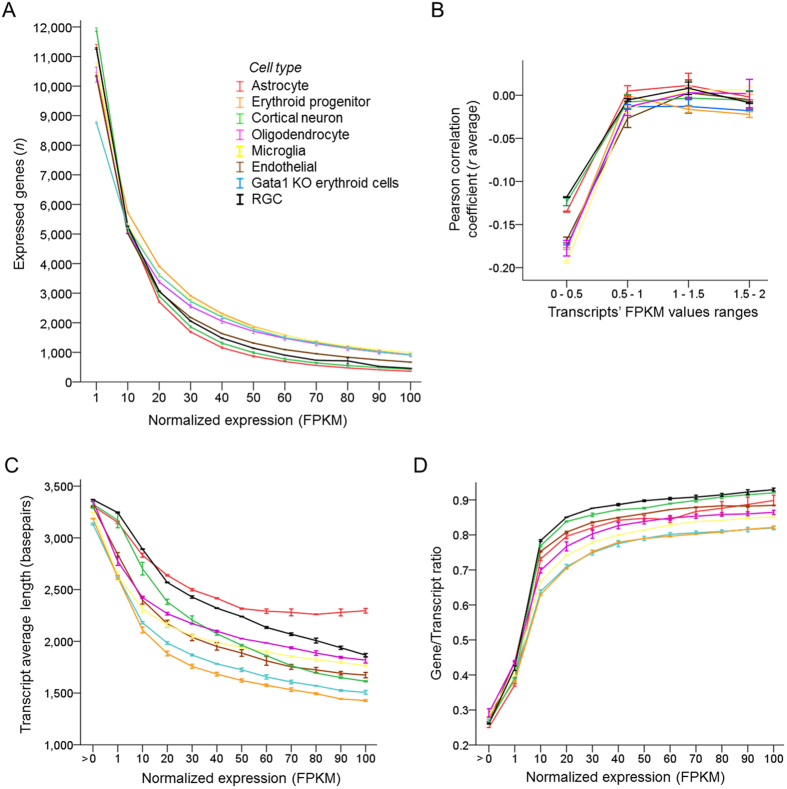
Cell types differ in the proportion of highly expressed genes and in the number of transcripts expressed per gene. (**A**) Number of expressed genes plotted across increasing normalized expression (FPKM) thresholds for different cell types, as marked (8 cell types, 2 replicates each, mean ± SEM shown; the mean FPKM values were statistically significantly different, *p* < 0.001, *F* = 63.2, by ANOVA with repeated measures, sphericity assumed). (**B**) Correlation analysis of transcript length and its level of normalized expression at different FPKM ranges shows weak correlation at very low levels of expression, but no correlation above 1 FPKM (shown Pearson correlation coefficient *r* mean ± SEM for each cell type, as marked; 2 replicates per cell type). (**C**) Cell type samples vary in average transcript length, which decreases at higher expression thresholds (shown transcript length mean ± SEM for each cell type, as marked; 2 replicates per cell type). (**D**) Cell type samples vary in ratio of expressed transcripts per gene (genes divided by transcripts), which increases at higher expression thresholds (shown transcript length mean ± SEM for each cell type, as marked; 2 replicates per cell type).

We then analyzed cell types’ expression profiles clustering (Fig. 2). Due to transcript length bias and possible noise at very low levels of expression (Fig. 3B), only genes expressed above 1 FPKM in at least one sample were retained for this analysis. Hierarchical cluster analysis segregated cell types into 3 groups (Fig. 2): (a) mesodermal origin myeloid precursors-derived MEPs and ECPs Gata1 KO; (b) although microglia also originated from the myeloid precursors they formed a discrete group on its own consistent with their divergence towards a different cell fate; and (c) neuroectodermal origin/neural stem cell-derived RGCs, cortical neurons, astrocytes, and oligodendrocytes, although endothelial cells also associated with this neuro-cluster despite their mesodermal origin. In the original study from which we obtained the raw reads for several of the cell types, the endothelial cells also clustered closely with some neural lineage cell types^14^. Thus, cell types’ expression profile clusters segregate consistently with their developmental lineages, cell fates, and previous analyses.

**Figure 4:**
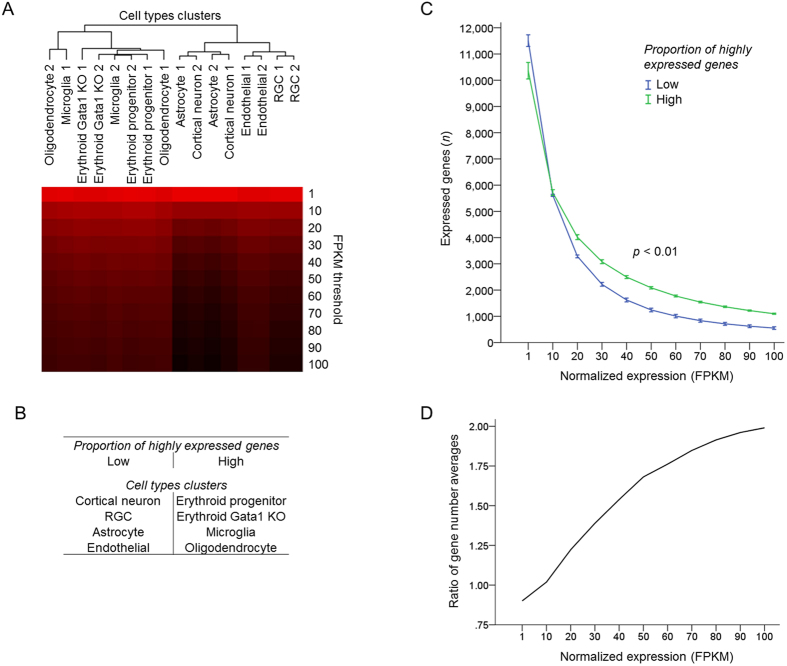
Cell types differing in the proportion of highly expressed genes segregate into clusters. (**A**) Dendrogram of cell types segregated into two upper hierarchy clusters based on proportion of highly expressed genes, by hierarchical clustering analysis of number of expressed genes at increasing normalized expression (FPKM) thresholds for different cell types. Uncentered Pearson correlation and centroid linkage with Gene Cluster 3.0 and visualization with Java Treeview 1.1.6r4. Number of genes is color coded, ranging from high (red) to low (black). (**B**) Summary of cell types’ clusters segregated in C based on patterns in proportion of highly expressed genes. (**C**) Number of expressed genes plotted across increasing normalized expression (FPKM) thresholds for the two groups of cell types that segregated in B based on proportion of highly expressed genes, as marked (2 groups, 4 cell types x 2 replicates each, mean ± SEM shown; *p* < 0.01 by ANOVA with repeated measures, posthoc LSD). (**D**) Ratio of expressed genes number averages in groups with high to low proportion of highly expressed genes (i.e., average number of genes expressed at a certain FKPM threshold in cell types comprising each cluster is used for ratio calculation), plotted across increasing normalized expression (FPKM) thresholds.

Next, we compared the number of genes expressed at different expression thresholds in cell types’ transcriptome profiles. We plotted the number of expressed genes across increasing normalized expression (FPKM) thresholds, and found that cell types differed significantly in the proportion of highly expressed genes (*p* < 0.001 by ANOVA with repeated measures, sphericity assumed, Fig. 3A), particularly ≥20 FPKM (also see later, Fig. 4C). We also tested with the upper quartile normalization and found similar differences between cell types in the proportion of highly expressed genes (*p* < 0.001, Figure S2), with the same four cell types comprising either upper or lower ranking groups (Table S1), as we also show later in Fig. 4B, although there were minor differences within the upper ranking group (Table S1). These data show that findings were not driven by the normalization method. Across samples, transcript length correlated weakly with the expression level at very low levels of expression, but there was no correlation above 1 FPKM (Fig. 3B). The differences in average transcript length at higher expression thresholds (≥1 FPKM, Fig. 3C) did not follow the pattern of how cell types differed in the proportion of highly expressed genes. For example, oligodendrocyte and microglia were amongst the cell types with the highest proportion of highly expressed genes, but both were at the middle of distribution of cell types’ average transcript length at high expression thresholds. We then examined whether cell types vary in the number of alternately spliced transcripts expressed from a locus at different expression thresholds. We found that while at low expression levels (<1 FPKM) the ratio of transcripts per gene was similar across cell types, at higher expression thresholds (≥1 FPKM) the ratio differed between cell types (Fig. 3D). Further, the differences between cell types in the ratio of transcripts per gene at high expression thresholds (particularly ≥20 FPKM) followed the pattern of differences between cell types in proportion of highly expressed genes (also ≥20 FPKM). These data suggest that cell types differ in proportion of highly expressed genes, and that these differences are associated with the number of alternatively spliced transcripts expressed per gene. Thus, our analyses show that cell types that express more variants of alternatively spliced transcripts per gene also tend to express higher proportion of highly expressed genes, suggesting that alternative splicing activity and the level of gene expression are linked.

Then we asked whether cell types segregate into groups based on patterns in proportion of highly expressed genes. Hierarchical cluster analysis segregated cell types into 2 major groups (Fig. 4A,B): (a) RGCs, astrocytes, cortical neurons, and endothelial cells and (b) MEPs, ECPs Gata1 KO, microglia, and oligodendrocytes. Similarly to clustering based on genes’ expression level (Fig. 2), the neuroectodermal origin neural stem cell-derived RGCs, cortical neurons, and astrocytes, as well as mesodermal-derived endothelial cells, clustered together. Further, mesodermal origin myeloid precursors-derived MEPs, ECPs Gata1 KO, and microglia clustered together, despite that in clustering based on genes’ expression level microglia formed a discrete group on its own. However, oligodendrocytes did not follow either the pattern of clustering based on genes’ expression level nor developmental lineage, as they clustered with mesodermal instead of their neuroectodermal origin cell types. These data suggests that differences between cell types in proportion of highly expressed genes represents a distinct property of the transcriptome that is related to, but is not always explained by, clustering based on genes’ expression levels and developmental lineage.

**Figure 5:**
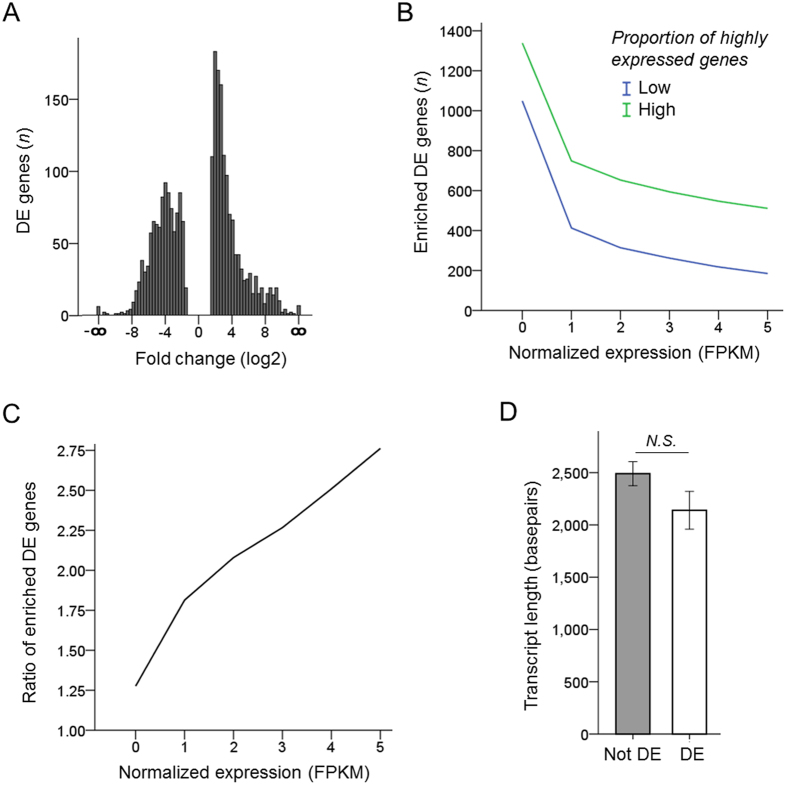
Differential expression analysis. (**A**) Frequencies of genes differentially expressed (DE) at different fold changes, with Cuffdiff DE *q*-values < 0.05, expression above 5 FPKM in at least one condition, and minimum 3 fold difference. Up-/down-regulation in a group with high proportion of highly expressed genes relative to a group with low proportion of highly expressed genes. (**B**,**C**) Number of DE genes enriched in groups with high or low proportion of highly expressed genes, as marked, plotted across increasing FPKM thresholds (**B**), and ratio of enriched DE genes numbers in groups with high to low proportion of highly expressed genes, plotted across increasing FPKM thresholds (**C**). Shown DE genes criteria: fold-change ≥3, CuffDiff *q*-value ≤ 0.05, and expression ≥ FPKM threshold in every cell type comprising a group. (**D**) Average length of not DE and DE transcripts is not significantly different. Not DE transcripts selected based on expression above 1 FPKM, with FPKM value within 1% of each other, and Cuffdiff DE *q-*value > 0.05. DE transcripts selected based on expression above 1 FPKM in at least one condition, fold change 2 or above, and Cuffdiff DE *q*-value < 0.05 (mean ± SEM shown; independent samples *t*-test, *N.S*. = not significant).

Next, we identified genes differentially enriched in the two clusters which segregated based on patterns in proportion of highly expressed genes. Cell types in each group were treated as one condition, and the analysis of differential expression between the two conditions was performed as above (see Methods for details). The difference between these groups in the average proportion of highly expressed genes was significant (*p* < 0.01; Fig. 4C). Further, the ratio of expressed genes number averages in groups with high to low proportion of highly expressed genes increases at higher expression thresholds (Fig. 4D). Consistent with one of the two groups of cell types having a higher proportion of highly expressed genes, more genes were differentially enriched in this group (Fig. 5A,B), and the ratio of enriched DE genes numbers in groups with high to low proportion of highly expressed genes also increased at higher expression thresholds (Fig. 5C).

Finally, we analyzed functional annotations of the DE genes. As we found that even weak correlation between the transcript length and expression level does not persist at expression above 1 FPKM in our filtered datasets (Fig. 1D), we set the expression threshold to be above 1 FPKM (in the condition in which its expression was enriched). We set the minimum fold-change threshold to 2. There was no significant difference between the average length of expressed DE and not-DE transcripts (Fig. 5D). We then proceeded to Functional Annotation Clustering of the biological processes GO terms using the Database for Annotation, Visualization and Integrated Discovery (DAVID), where higher enrichment score signifies more cluster enrichment and is the geometric mean (in -log scale) of *p*-values for the individual annotation categories comprising the cluster^26^,^27^. We found enrichment of biological functions involved in negative regulation of gene expression in the group of cell types with low proportion of highly expressed genes, and an enrichment of biological functions involved in regulation of transcription and RNA splicing in the group of cell types with high proportion of highly expressed genes (Tables 3, S2 and S3). Our analyses raise the hypothesis that the genes comprising these predicted biological pathways underlie the intrinsic differences between cell types in proportion of highly expressed genes and the number of alternatively spliced transcripts expressed per gene.

## Discussion

The molecular mechanisms of how cells regulate balance in global properties of the transcriptome are not well understood, and it is unknown whether various mammalian cell types differ in their homeostatically maintained transcriptome properties. Broadly speaking, homeostasis could be regulated at the level of transcription, stabilization, and degradation, as well as alternative promoter site usage and mRNA splicing. Prior studies attempted to decipher how cells maintain global properties of the transcriptome in a stable state by investigating the molecular mechanisms controlling synthesis and degradation of RNA, the equilibrium between these processes, and the thermodynamic models explaining the transcriptome homeostasis^1^,^2^,^3^,^4^,^5^,^6^,^7^,^8^,^9^,^10^,^11^,^12^.

Here we investigated whether various mammalian cell types differ in global transcriptome properties. To address this question, we compared 8 mouse cell types’ RNA-seq datasets. All cell types were acutely purified primary cells, except ECPs Gata1 KO, which were a cell line derived from immature embryonic mouse erythroblasts with targeted Gata1 gene deletion^15^,^28^. However, despite ECPs Gata1 KO being a cell line, it was most closely associated on all parameters with acutely purified MEPs^15^, consistent with their erythroid precursor lineage, suggesting that ECPs Gata1 KO being a cell line or lacking the ability to differentiate into the erythroid cells due to the absence of Gata1 did not substantially alter its global transcriptome properties. We found that different cell types vary in proportion of highly expressed genes and the number of alternatively spliced transcripts expressed per gene, and that the cell types that express more variants of alternatively spliced transcripts per gene are those that have higher proportion of highly expressed genes. Such association could occur if, for example, the cell types with higher proportion of highly expressed genes would have elevated basal transcriptional activity, which also involves splicing activity, and result in both of these global parameters to be higher in the same cell types. Remarkably, cell types segregated into two upper hierarchy clusters based on high or low proportion of highly expressed genes alone. Although clustering was associated with cell types’ developmental lineage for most cell types, because it was not associated with all cell types that we tested, the proportion of highly expressed genes alone would not be sufficient for establishing cell types identities. However, this property of transcriptome may not be determined by the developmental lineage alone, but also by other factors, such as positioning in the tissue and signaling by adjacent cells. With regards to the highly expressed genes themselves, since they may have stronger weight in clustering analysis, it would be interesting to investigate in future studies the extent to which the underlying biology may be driven specifically by these groups of genes.

Are there consistent differences between the specific genes or pathways expressed by cells based on proportion of highly expressed or more highly spliced genes? Analysis of Functional Annotation Clustering of the GO terms associated with genes differentially enriched in the two clusters of cell types identified pathways involved in regulating gene expression and RNA splicing. Thus, cell types could vary in intrinsic properties of the transcriptome by maintaining different proportion of highly expressed genes and different number of alternatively spliced transcripts expressed per gene. These processes, in turn, may reflect intrinsic differences between cell types in coordination of synthesis, splicing, and degradation of RNA molecules. This discovery should promote investigation into contributions of individual genes’ or pathways’ effects on the transcriptome homeostasis and subsequent downstream cellular or tissue phenotypes. The additional identified GO terms may also be involved in these biological processes and could provide clues for future studies.

What is the biological significance of spatio-temporal variance between cell types in the proportion of low and highly expressed genes and the number of alternatively spliced transcripts expressed per gene? More highly expressed genes exhibit more gradients in their concentration in cells or tissues, which could lead to more fine-tuned interactions and increased functional complexity in the downstream molecular network. This increased functional complexity could underlie differences between cell types at different stages in development or at different positions within the tissue, much like gradients in morphogenic factors during development contribute to anatomical complexity of an organ. A higher number of alternatively spliced transcripts expressed per gene may also enable increased functional complexity stemming from that gene locus. Because we find that cell types that express more variants of alternatively spliced transcripts per gene are those that demonstrate a higher proportion of highly expressed genes, these properties could be coupled and involved in regulation of the same underlying biological attribute(s). However, a higher number of low expressed genes may also lead to more fine-tuned regulation and increased functional complexity, if they are not regarded by the cell as noise. It is also possible that a high proportion of highly expressed genes may be indicative of a larger total transcriptome size^29^, and may be related to cell volume and cellular metabolism, which interestingly was one of the biological processes enriched in cell types with higher proportion of highly expressed genes (Table 3). These hypotheses need to be addressed experimentally in future studies.

Our observations have a unique implication for RNA-seq studies where transcriptional or epigenetic factors are experimentally targeted, as such factors may regulate global properties of the transcriptome. For example, if transcriptional or epigenetic factor manipulations elicit a negative feedback mechanism to downregulate highly expressed genes or the frequency of RNA splicing events, they will also render differential gene expression analysis difficult to interpret. While identifying absolute levels of gene expression requires additional methods such as synthetic spike-in standards^30^, analyzing proportion of highly expressed genes and the number of alternatively spliced transcripts expressed per gene could be done with RNA-seq data generated using standard methods, which will at least enable accounting for such relative differences. Utilizing spike-in standards^30^ in future studies is also important because it will facilitate investigating various aspects of the transcriptome biology alluded to by our studies. For example, one could then derive a more accurate reconstruction of alternatively spliced transcripts that are expressed at a very low level, as well as predict the negative feedback threshold for global homeostatic downregulation of highly expressed genes, which our studies suggest may differ between cell types and possibly between species.

In conclusion, our findings suggest that cell types vary in intrinsic properties of the transcriptome by maintaining different proportion of highly expressed genes and different number of alternatively spliced transcripts expressed per gene. Such intrinsic differences between cell types could be associated with differential coordination of synthesis, splicing, and degradation of RNA molecules, and should be accounted for in comparative RNA-seq analysis, particularly if transcriptional or epigenetic factors are experimentally targeted. The molecular mechanisms and pathways regulating global properties of transcriptome, their biological significance, and the differences between more of the various cell types and of the same cell type between species, are important to investigate in future studies.

## Methods

### Cell purification methods and RNA-seq datasets Gene Expression Omnibus (GEO) accessions

Astrocytes were purified by FACS from single cell suspension cortices of Aldh1l1–BAC-eGFP transgenic mice following an established protocol^14^ (original raw reads available from the NCBI GEO accession numbers GSE52564/GSM1269903/GSM1269904). Endothelial cells were purified by FACS from single cell suspension cortices of Tie2–EGFP transgenic mice following an established protocol^14^ (original raw reads available from the NCBI GEO accession numbers GSE52564/GSM1269915/GSM1269916). Cortical neurons were purified from mice cortices single cell suspension by immunopanning for L1CAM after depletion of endothelial cells, oligodendrocyte precursor cells, microglia and macrophages (using BSL1, O4, and CD45, respectively), and washing off the nonadherent cells, following an established protocol^14^ (original raw reads available from the NCBI GEO accession numbers GSE52564/GSM1269905/GSM1269906). Oligodendrocytes were purified from mice cortices single cell suspension by immunopanning for MOG after depletion of endothelial cells, oligodendrocyte precursor cells, microglia and macrophages (using BSL1, PDGFRα, A2B5, and CD45, respectively), and washing off the nonadherent cells, following an established protocol^14^ (original raw reads available from the NCBI GEO accession numbers GSE52564/GSM1269911/GSM1269912). Microglia were purified from mice cortices single cell suspension by immunopanning for CD45 after depletion of macrophages through perfusing the mice with PBS to wash away blood from the brain, following an established protocol^14^ (original raw reads available from the NCBI GEO accession numbers GSE52564/GSM1269913/GSM1269914). Megakaryocyte-erythroid progenitors (MEP) were purified from adult mouse bone marrow by FACS^15^ using an established protocol [Lineage(−), cKit(+), Sca1(−), CD34low, CD16/32(−)]^31^ (original raw reads available from the NCBI GEO accession numbers GSE40522/GSM995525). Erythroid-committed precursors (ECP) Gata1 KO (which cannot differentiate into the erythroid cells without Gata1) were derived from immature embryonic mouse erythroblasts with targeted Gata1 gene deletion^15^ using an established protocol^28^ (original raw reads available from the NCBI GEO accession numbers GSE40522/GSM995536). Retinal ganglion cells (RGCs) were purified by authors from postnatal day 5 mice eyes single cell suspension by immunopanning for Thy1 (CD90, MCA02R, Serotec) after depletion of macrophages (using anti-mouse macrophage antibody, AIA31240, Accurate Chemical) and washing off the nonadherent cells, following an established protocol^13^,^32^, and RNA extracted using the Direct-zol RNA kit (Zymo Research) had a RIN ≥ 8.5 (Bioanalyzer 2100, Agilent 6000 kit; raw reads available from the NCBI GEO accession numbers *pending*). All animal procedures for collecting RGCs were approved by the University of Miami Institutional Animal Care and Use Committee and by the Institutional Biosafety Committee at the University of Miami, and performed in accordance with the ARVO Statement for the Use of Animals in Ophthalmic and Visual Research. C57BL/6J mice were obtained from Charles River Laboratories, Inc. For all cell types samples libraries were prepared using polyA-selected RNA and paired reads sequenced 100 bp from each end on HiSeq 2000 Sequencer (Illumina)^14^,^15^. All cell types samples included two biological replicates for which raw reads and analyzed/reanalyzed datasets are available through the GEO accession numbers provided above.

### RNA-seq analysis pipeline commands and software versions

Reads were mapped to mouse reference genome mm10 (UCSC Genome Browser) and a comprehensive transcriptome annotation database GTF file, which was assembled by using the UCSC Table Browser Intersection utility to merge the GENCODE M4^33^ transcripts in a non-redundant manner with the UCSC Gene Track^34^ transcripts that did not overlap more than 90% with the GENCODE transcripts. The raw reads were mapped using the TopHat/Bowtie2/Cufflinks pipeline^17^,^18^,^19^, with -g option, to construct merged GTF file that included the annotated and novel transcript structures from all samples. We then used the IntersectBed tool (Bedtools) to retain only the reads that mapped to the merged GTF, which was converted to BED with Gtf2bed tool (Bedops). This filtering step allowed selecting the reads which contributed to the identified gene structures, and exclude noise and artifacts even if they mapped to the genome but did not contribute to gene structure. Next, we selected only uniquely mapped and properly paired reads using View -bq 4 -bh -f2 -F12 command (Samtools). After this step we used DownsampleSam tool (Picard) to randomly subsample equal number of paired reads, which provided representative samples of the same size for all samples (34.6 M per sample/replicate; properly paired and total reads count with Flagstat, Samtools). Then we used the TopHat/Bowtie2/Cufflinks/Cuffdiff pipeline^17^,^18^,^19^ with -g option for determining normalized expression in fragments per kilobase of transcript sequence per million mapped fragments (FPKMs) in each replicate of each sample with Cuffdiff’s across-sample normalization (Table 2), and assessed the filtered reads aligned to transcripts or introns and intergenic regions using RnaSeqMetrics (http://broadinstitute.github.io/picard)^35^. For the differential expression analysis where cell types in each of the two upper hierarchy clusters were treated as one condition, each replicate of each cell type was assigned to one of only two cluster groups. For differential expression analysis, the Cuffdiff *q*-value (which is the FDR corrected *p*-value^17^,^18^) cut off was set to 0.05. For the upper quartile normalization, the FPKMs were normalized to the upper quartile across samples and scaled by the mean of upper quartiles from all samples. Software versions used: Tophat 2.0.12, Bowtie 2.2.4, Cufflinks 2.2.1, Samtools 0.1.19, Picard 1.79, Bedops 2.4.2, Bedtools 2.19.0. Analyses were performed on the Orchestra High Performance Compute Cluster at Harvard Medical School NIH supported shared facility, consisting of thousands of processing cores and terabytes of associated storage. The datasets from these analyses are available through the GEO accession Series GSE85458.

### Statistics, Cluster analysis, and Functional Annotations

Pearson correlation and matrix analysis (2-tailed) of gene expression profiles, as well as ANOVA with posthoc LSD, were preformed using SPSS software with *p* < 0.05 indicating statistical significance. Dendrogram and hierarchical clustering heat maps, with uncentered Pearson correlation and centroid linkage, were generated using Gene Cluster 3.0 and visualized with Java Treeview 1.1.6r4^36^,^37^. Functional Annotation Clustering of the GO terms associated with differentially expressed genes was performed using the Database for Annotation, Visualization and Integrated Discovery (DAVID), with higher enrichment score signifying more cluster enrichment^26^,^27^. Enrichment score is the geometric mean (in −log scale) of *p*-values for the individual annotation categories comprising a cluster^26^,^27^. Minimum enrichment score threshold was set to 0.75, and clusters implicated in the same higher order biological process were manually merged and the averages of their enrichment scores are shown.

## Additional Information

**How to cite this article**: Trakhtenberg, E. F. *et al*. Cell types differ in global coordination of splicing and proportion of highly expressed genes. *Sci. Rep.* **6**, 32249; doi: 10.1038/srep32249 (2016).

## Supplementary Material

Supplementary Information

## Figures and Tables

**Table 1:** Sources of the cell type-specific RNA-seq datasets used in this study.

Cell Type	Data Source
Retinal ganglion cells	Authors, Trakhtenberg *et al*.^13^
Cortical neurons	Zhang *et al*.^14^
Astrocytes	Zhang *et al*.^14^
Oligodendrocytes	Zhang *et al*.^14^
Microglia	Zhang *et al*.^14^
Endothelial cells	Zhang *et al*.^14^
Megakaryocyte-erythroid progenitors	Paralkar *et al*.^15^
Erythroid-committed precursors Gata1 KO	Paralkar *et al*.^15^

**Table 2:** Correlation Matrix (Pearson, 2-tailed).

	RGC 1	RGC 2	Cortical neuron 1	Cortical neuron 2	Astrocyte 1	Astrocyte 2	Oligodendrocyte 1	Oligodendrocyte 2	Microglia 1	Microglia 2	Endothelial cell 1	Endothelial cell 2	ECP Gata1KO 1	ECP Gata1 KO 2	MEP 1	MEP 2
RGC 1	1															
RGC 2	**0.98**	1														
Cortical neuron 1	0.41	0.46	1													
Cortical neuron 2	0.70	0.75	**0.88**	1												
Astrocyte 1	0.40	0.42	0.43	0.56	1											
Astrocyte 2	0.39	0.41	0.36	0.50	**0.98**	1										
Oligodendrocyte 1	0.52	0.57	0.51	0.64	0.39	0.34	1									
Oligodendrocyte 2	0.49	0.57	0.64	0.73	0.43	0.36	**0.97**	1								
Microglia 1	0.14	0.14	0.15	0.22	0.46	0.46	0.15	0.17	1							
Microglia 2	0.11	0.11	0.14	0.20	0.43	0.43	0.12	0.14	**0.99**	1						
Endothelial cell 1	0.49	0.53	0.58	0.69	0.41	0.38	0.55	0.59	0.28	0.23	1					
Endothelial cell 2	0.51	0.52	0.46	0.62	0.37	0.36	0.53	0.53	0.28	0.24	**0.98**	1				
ECP Gata1 KO 1	0.25	0.30	0.26	0.36	0.19	0.18	0.26	0.29	0.16	0.15	0.40	0.38	1			
ECP Gata1 KO 2	0.25	0.26	0.22	0.32	0.17	0.17	0.23	0.23	0.18	0.17	0.40	0.41	**0.93**	1		
MEP 1	0.27	0.28	0.21	0.31	0.20	0.20	0.21	0.21	0.19	0.18	0.40	0.41	0.85	0.88	1	
MEP 2	0.27	0.34	0.41	0.48	0.26	0.24	0.31	0.38	0.19	0.18	0.48	0.43	0.90	0.82	**0.88**	1

Replicates are highly correlated within but not between the samples.

**Table 3:** Functional Annotation Clustering of the GO terms using DAVID.

Functional Annotation Cluster	Enrichment Score	Number of genes
*Cell types cluster with low proportion of highly expressed genes*		
Regulation of neurotransmitter signaling	0.95	8
Regulation of phosphorylation	0.94	6
Negative regulation of gene expression	0.85	7
*Cell types cluster with high proportion of highly expressed genes*		
Protein transport and nuclear import	2.87	18
Cellular metabolism	1.02	32
Cation and pH homeostasis	1.02	3
Cell cycle	0.81	10
Cellular response to nutrient levels	0.78	3
Regulation of transcription and RNA splicing	0.75	15

The analysis showed differential enrichment of biological functions involved in regulating gene expression and other cellular processes in cell types clusters with low or high proportion of highly expressed genes. Minimum Enrichment Score threshold was set to 0.75. Clusters implicated in the same higher order biological process were manually merged (e.g., metabolic processes of nucleobase, alkaloid, oxidoreduction coenzyme, cellular amide, and membrane lipid, were merged under Cellular Metabolism category post hoc) and the averages of their enrichment scores are shown.
