# Supplementary material for: Cell types differ in global coordination of splicing and proportion of highly expressed genes
Source: Sci Rep. 2016 Aug 31;6:32249. doi: 10.1038/srep32249 (PMC5006053; doi:10.1038/srep32249)
Supplement: Supplementary Information [file srep32249-s1.pdf]

SUPPLEMENTARY INFORMATION

TITLE PAGE

Cell types differ in global coordination of splicing and proportion of highly expressed genes

Ephraim F. Trakhtenberg[1]*, Nam Pho[2], Kristina M. Holton[2], Thomas W. Chittenden[2], Jeffrey L. Goldberg[3], and Lingsheng Dong[2].

1. Department of Neurosurgery, F.M. Kirby Neurobiology Center, Boston Children's Hospital, Harvard Medical School, Boston, MA; 2. Research Computing Group, Harvard Medical School, Boston. MA. 3. Byers Eye Institute, Stanford University, Palo Alto, CA.

*Correspondence should be addressed to Dr. Ephraim F Trakhtenberg, CLSB 13072, Boston Children's Hospital, 300 Longwood Avenue, Boston, MA 02115; Email: Ephraim.Trakhtenberg@childrens.harvard.edu

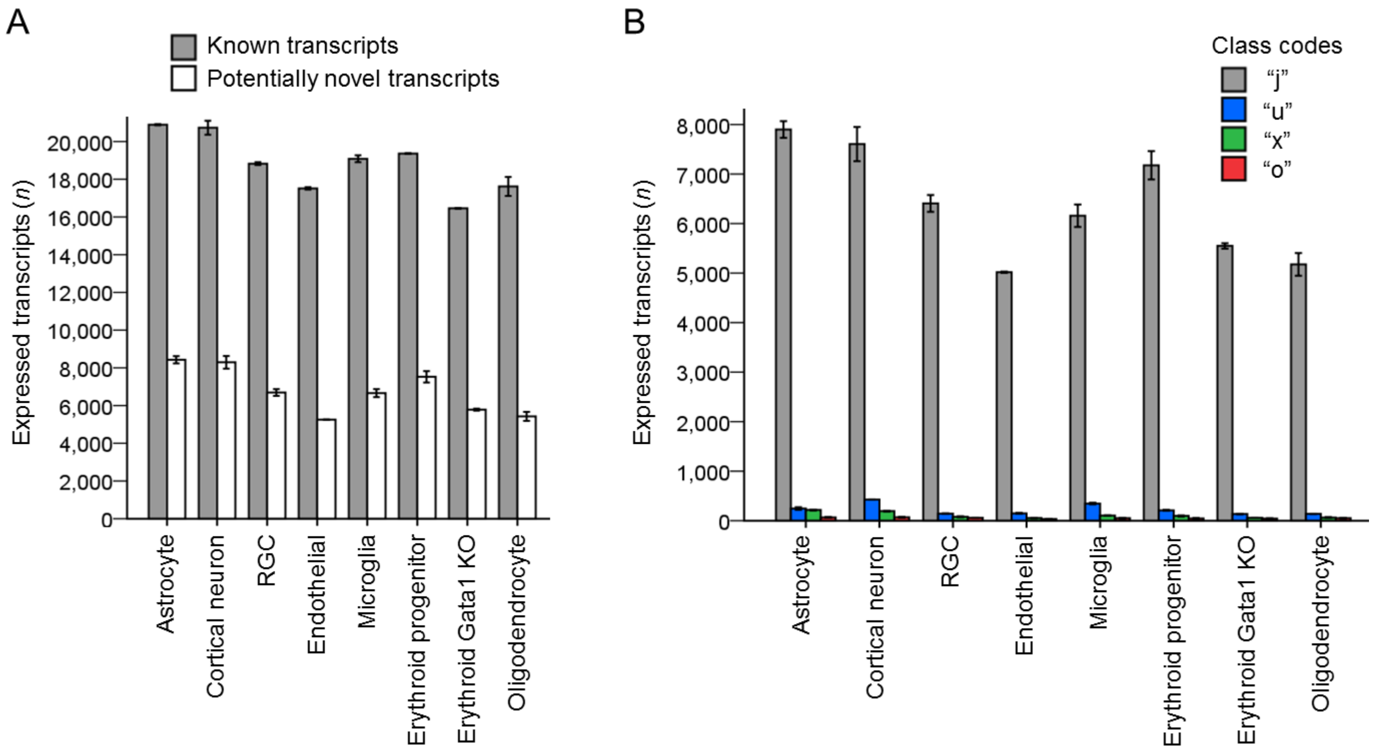

Figure S1. **Transcripts by Cuffcompare class code classification across cell types**. (**A**) Number of transcripts detected at 1 FPKM expression threshold, classified as either known (class code "=") or potentially novel (class codes "j", "u" (a.k.a. "-"), "x", "i", and "o"). Other class codes were not detected (2 replicates per cell type, mean ± SEM shown). (**B**) Class codes distribution of the potentially novel transcripts in *A*: "j", at least 1 novel and 1 known splice junction; "u" (a.k.a. "-"), novel transcript in the intergenic region; "x", exonic overlap with known antisense exon; "o", partial exonic overlap with known exon; and "i", exon within known intron (ranged from 0 to 3 transcripts per sample and bar could not be seen). Other class codes were not detected.

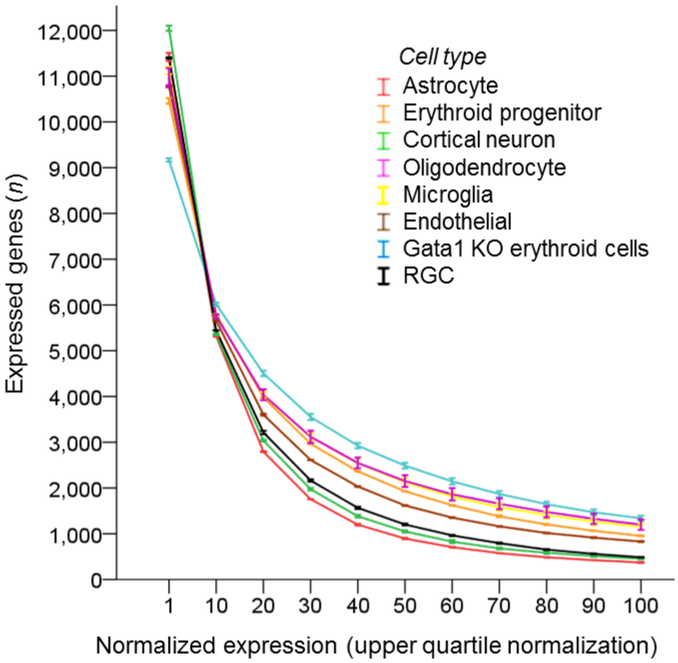

Figure S2. **Cell types differ in the proportion of highly expressed genes using upper quartile normalization**. Number of expressed genes plotted across increasing normalized expression (upper quartile normalization) thresholds for different cell types, as marked (8 cell types, 2 replicates each, mean ± SEM shown; the means were statistically significantly different, $p < 0.001$, $F = 63.2$, by ANOVA with repeated measures, sphericity assumed).

Table S1. **Ranking of cell types by the proportion of highly expressed genes using FPKM and upper quartile normalization**. *Left*, ranking of the cell types by the proportion of highly expressed genes using FPKM normalization (ranking shown for 90, and is the same for 100, see thresholds in Figure 3A), or *right,* by upper quartile normalization (ranking shown for 90, and is the same for 100, see thresholds in Figure S1). Ranking: higher to lower position in the column indicates higher to lower proportion of highly expressed genes.

	FPKM normalization	*Upper quartile normalization*
Upper half	Microglia	Gata1 KO erythroid cells
	Erythroid progenitor	Oligodendrocyte
	Oligodendrocyte	Microglia
	Gata1 KO erythroid cells	Erythroid progenitor
Lower half	Endothelial	Endothelial
	RGC	RGC
	Cortical neuron	Cortical neuron
	Astrocyte	Astrocyte

Table S2. **Biological processes enriched in the group of cell types with low proportion of highly expressed genes**. Enrichment scores are shown.

Functional Annotation Cluster	Regulation of neurotransmitter signaling	Regulation of phosphorylation	Negative regulation of gene expression
Enrichment Score	0.95	0.94	0.85
Genes #	8	6	7
RAB3A	+		
SYN3	+		
UNC13B	+		
RAB3A	+		
SYN3	+		
PAFAH1B1	+		
AGRN	+		
UNC13B	+		
ACVR2A		+	
SPAG9		+	
CCDC88A		+	
GLMN		+	
APLP2		+	
TRIB2		+	
HES5			+
ZRANB1			+
TXN1			+
BHLHE40			+
TRIM24			+
DNAJB5			+
C1D			+

Table S3. **Biological processes enriched in the group of cell types with high proportion of highly expressed genes**. Clusters implicated in the same higher order biological process were manually merged and averages of their enrichment scores are shown (e.g., metabolic processes of nucleobase, alkaloid, oxidoreduction, cellular amide, and membrane lipid, were merged under Cellular Metabolism category).

Functional Annotation Cluster	Protein transport and nuclear import	Cellular metabolism	Cation and pH homeostasis	Cell cycle	Cellular response to nutrient levels	Regulation of transcription and RNA splicing
Enrichment Score	2.87	1.02	1.02	0.81	0.78	0.75
Genes #	18	32	3	10	3	15
DERL1	+	+				
RFFL	+	+				
TRP53	+		+		+	+
AP2S1	+					
ARAP1	+					
DNHD1	+					
FAM53A	+					
GIPC1	+					
HPS4	+					
MTX1	+					
NUTF2	+					
POLA2	+					
RAN	+			+		
SNX2	+					
STX2	+					
TIMM13	+					
TOMM20	+					
XPO6	+					
ASPDH		+				
TALDO1		+				
NCF2		+				
PGD		+				
DCXR		+				
ALDOA		+				
HK1		+				
GAPDH		+				
LONP1		+				
NUDT9		+				
AHCY		+				
BCAT2		+				
AFMID		+				
URM1		+				
KDM2B		+				
ATG7		+				
FBXW4		+				
UCHL5		+				
RBCK1		+				
RNF19B		+				

(*Table continues*)

Table S3. Continued

	Protein transport and nuclear import	Cellular metabolism	Cation and pH homeostasis	Cell cycle	Cellular response to nutrient levels	Regulation of transcription and RNA splicing
FEM1A		+				
ASB6		+				
TRIM11		+				
DHODH		+				
AMPD2		+				
GART		+				
SPHK2		+				
CLN3		+	+		+	
HEXB		+				
CLN8		+				
MAFG			+			
CDC123				+		
FOXO4				+		
TCF3				+		+
LZTS2				+		
ARHGEF2				+		
SIRT2				+		
SETD8				+		
HAUS8				+		
PES1				+		
CTSD					+	
PRPF31						+
TRA2B						+
PRMT5						+
LSM2						+
MBNL1						+
ZBTB7B						+
MEN1						+
TAF6L						+
CAMTA2						+
FUBP3						+
NFYA						+
CARD9						+
IRAK1						+
